# Beyond a Gossypiboma

**DOI:** 10.1155/2012/263841

**Published:** 2012-08-05

**Authors:** Abdul Haque M. Quraishi

**Affiliations:** Department of Surgery, Government Medical College, 22, Vijaynagar, Chhaoni, Nagpur 440013, India

## Abstract

Gossypiboma or a retained surgical sponge is a rare but avoidable surgical complication. It leads to considerable morbidity and at times even mortality. We report a case of a 24-year-old lady who presented one month after a Caesarean operation with complaints of fever, pain in abdomen, and vomiting. After the clinical examination a possibility of a retained surgical sponge was entertained. However a CT scan of abdomen revealed the complete diagnosis and helped in treating the patient surgically with a successful outcome. A review of the literature and all the relevant issues in the management of such a case have been discussed.

## 1. Introduction

Gossypiboma is a term used for a retained surgical sponge and is derived from gossypium (Latin cotton) and “boma” (Swahili place of concealment). Two usual responses lead to the detection of a retained sponge. The first type is an exudative inflammatory reaction with the formation of an abscess and usually leads to early detection and surgical removal. The second type is aseptic with a fibrotic reaction to the cotton material and development of a mass [1]. A gossypiboma may be associated with a bowel perforation which can be diagnosed preoperatively by a CT scan. An attempt to find associated complications of gossypiboma should be made to avoid missing them.

## 2. Case Report

 A 24-year-old lady presented to our hospital one month after a Caesarean operation at another institute. This was her second Caesarean operation and was done as an emergency. Two days later the patient developed a pain in abdomen and fever; she was discharged on the eighth day. The symptoms exacerbated along with distension of abdomen, for which she consulted a private hospital and received cefotaxime and metronidazole. She was also referred back to the hospital where she was operated upon and she was treated symptomatically. However her condition worsened and she was referred to our hospital. She complained of fever, pain in abdomen, and vomiting. She also complained of loose stools and mucus discharge per rectum. On examination she was febrile and had tachycardia. Her abdomen was tender and lower abdomen had a vague tender mass.

**Figure 1:**
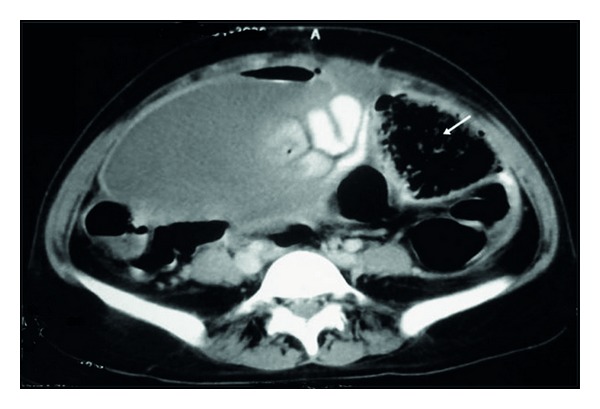
CT of abdomen showing a large fluid collection and another heterogeneous collection with air trapped within, on the left side [gossypiboma].

Her lab reports revealed neutrophilia and anaemia. A plain radiograph of abdomen was essentially normal. US of abdomen revealed a bulky subinvoluted uterus with a large collection within (pyometra), hypoperistaltic bowel loops, and mild hydroureteronephrosis on the right. However, the sonological impression of pyometra was not being correlated by gynaecological evaluation and hence a contrast-enhanced CT scan of abdomen was done (Figure 1). It revealed a mesh-like structure in the lower abdomen with air trapped within. An oral dye was administered during the procedure which revealed a leak in the small bowel. A diagnosis of gossypiboma with bowel perforation was made. After due preparation, the patient was posted for an exploratory laparotomy.

**Figure 2:**
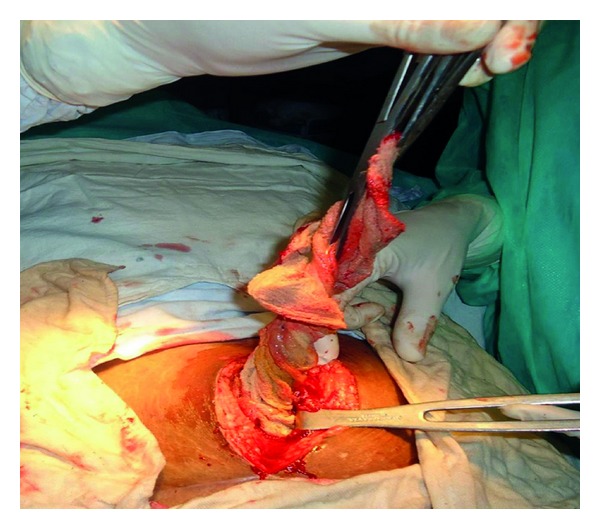
Intraoperative photograph showing the retained surgical sponge being removed.

**Figure 3:**
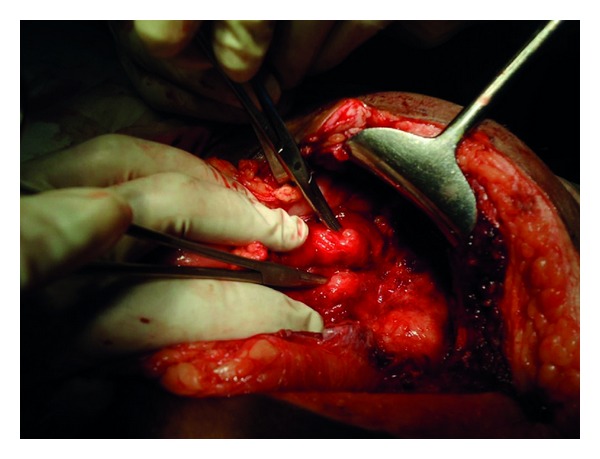
Two small bowel perforations being demonstrated by haemostats.

Abdomen was accessed through a midline vertical incision. On exploration a retained surgical sponge was found along with one litre of pus lying in a walled off cavity in the lower abdomen. The retained surgical sponge was removed (Figure 2). The pus was sucked out. A pus sample was sent for culture and antibiotic sensitivity which was later reported to have grown *Escherichia coli*. There were multiple dense adhesions of the small bowel. On careful separation of the adhesions two ileal perforations were seen (Figure 3). These perforations were half a centimetre in size and 40 cm apart. The intervening bowel was edematous. A resection of the perforation bearing bowel was done followed by anastomosis. The resected specimen was sent for histopathology which later revealed features of acute inflammation, and no granulomas were seen.

The patient did well after the surgery. There was infection of the lower part of the abdominal wound, which was left open and a dressing was done regularly. A secondary suturing was done two weeks after the surgery and the patient was discharged.

The hospital where the original surgery was done was informed to facilitate initiation of preventive steps. An inquiry into the details of the previous surgery revealed that the operation was done as an emergency and that it was difficult because of bowel adhesions due to previous Caesarean operation. The closure of abdomen was done by junior residents. A proper procedure for surgical counts was also found to be lacking.

## 3. Discussion

It is estimated that a gossypiboma may occur in 1 in 1,000–1,500 intra-abdominal operations [2]. It was found that patients with retained foreign bodies were more likely to have had emergency surgery, an unexpected change in surgical procedure, or a higher mean body mass index and were less likely to have had sponge counts performed at the time of the operation [3]. 

A pyoperitoneum or an organised hematoma may present in a similar manner. Thus, a high index of suspicion is required. A plain radiograph may help in a case of a retained sponge with a radio-opaque marker. In our case the sponge had no such marker. A US study helps in the diagnosis by revealing cystic masses with central echogenic wavy stripes with acoustic shadows. A contrast-enhanced CT shows air trapped nonenhancing mass [4]. An upper gastrointestinal dye study along with CT helps in diagnosing perforation. There have been cases of missed perforations reported in the literature [5]. In our case, evidence of perforation was demonstrated on the CT preoperatively. One must investigate and find causes apart from the gossypiboma which may contribute to the clinical condition. In the present case the perforation may have resulted due to separation of adhesions during the Caesarean operation as the patient was symptomatic immediately after surgery. However, a retained sponge can itself lead to abscess formation and bowel perforation. It can also lead to fistulation and transmural migration into the alimentary tract. Such sponges may be expelled spontaneously. They can also be removed endoscopically [6]. Laparoscopy can also be employed in selected cases for retrieval of retained foreign bodies [7]. 

 Sponge, sharp, and instrument counts have been used as protection against this problem. Four separate counts have been recommended: the first when the instruments are set up or sponges unpackaged, a second before surgery begins, a third as closure begins, and the final count performed during skin closure. This practice of sponge counts is heavily dependent on human performance practices and is thus subject to human error [8]. Several adjunct technologies are under development for supporting surgical teams in performing counts and reducing instances of lost or retained sponges. One example is a barcode system, which accounts for sponges based on affixed, two-dimensional matrix labels [9]. Two additional technologies embed electronic chips within sponges: the electronic article surveillance (EAS) system, which uses magnetomechanical technology [10]; and radiofrequency identification (RFID) microchips, which receive signals sent by a wandlike handheld scanner and respond with unique identification code [11].

Medico legal aspects of negligence and compensation also have to be addressed during the management.
